# Association Between Physical Exercise and Mental Health During the COVID-19 Outbreak in China: A Nationwide Cross-Sectional Study

**DOI:** 10.3389/fpsyt.2021.722448

**Published:** 2021-08-16

**Authors:** Yingjun Nie, Yuanyan Ma, Yankong Wu, Jiahui Li, Ting Liu, Ce Zhang, Chennan Lv, Jie Zhu

**Affiliations:** ^1^College of the Arts, Wuhan Sports University, Wuhan, China; ^2^College of Science and Technology, Wuhan Sports University, Wuhan, China; ^3^Graduate School, Wuhan Sports University, Wuhan, China; ^4^College of Health Sciences, Wuhan Sports University, Wuhan, China

**Keywords:** COVID-19, Chinese residents, physical exercise, mental health, home quarantine

## Abstract

The COVID-19 has undergone several mutations, and caused deleterious effects on physical and mental health of people worldwide. Whilst physical exercise is known for its positive effect on enhancing immunity and reducing the negative consequences of unhealthy emotional states caused by the pandemic; there is a severe lack of psychological exercise intervention measures and mitigation strategies to advance the knowledge and role of physical exercise to improve mental health in most countries. This study surveyed the association between physical exercise and mental health burden during the COVID-19 outbreak in China to better understand the influence of different physical exercise types on reducing mental health burden during the pandemic. ANOVA, binary logistic regression, the chi-square test, and Spearman's correlation analysis were used for statistical analysis. 14,715 participants were included. The results showed that Chinese residents had several poor mental health conditions during the COVID-19 outbreak. And there was a significant positive correlation between the extent of adverse effects on mental health and provincial proportions of confirmed COVID-19 cases (r = 0.365, *p* < 0.05). Some main factors caused an unhealthy psychological status, including epidemic severity (62.77%, 95% CI 58.62-65.64%), prolonged home quarantine (60.84%, 95% CI 58.15-63.25%), spread of large amounts of negative information about COVID-19 in the media (50.78%, 95% CI 47.46-53.15%), limitations in daily life and social interaction (45.93%, 95%CI 42.46-47.55%), concerns about students' learning (43.13%, 95% CI 40.26-45.48%), and worries about being infected (41.13%, 95% CI 39.16-45.23%). There was a significant association between physical exercise and mental health. The largest associations were seen for home-based group entertainment exercise (i.e., family games, rope skipping, and badminton), Chinese traditional sports (i.e., Chinese martial arts, Taijiquan and Qigong), and popular sports (i.e., yoga, video dancing, sensory-motor games, and whole-body vibration), as well as durations of 30-60 min per session, frequencies of three to five times per week and a total of 120-270 min of moderate-intensity exercise weekly during the COVID-19 outbreak (*p* < 0.05).

## Introduction

The novel coronavirus disease (COVID-19) has led to over 180 million confirmed cases and over 4 million deaths globally as of 12th July 2021, including 92,066 confirmed cases and 4,636 deaths in China (1). Governmental in various countries have implemented urgent national containment strategies to prevent the spread of the pandemic and reduce the risk of national medical systems becoming critically overburdened (2–4). Although social distancing and home quarantine measures aimed to reduce human-to-human transmission of the COVID-19, such measures have caused dramatic changes in people's routine daily activities and lifestyles, e.g., decreased physical activity and increased sedentary time (5). The great life-altering may not only adversely affect the immune function leading to several chronic diseases (6), but also increase the risk of mental health problems (e.g., depression, anxiety, and loneliness) (7, 8) and even psychological imbalance and instability (9). For example, recent studies have shown that 54% of the general population had mental health burden and 29% had anxiety symptoms during the COVID-19 outbreak in China (10), almost 30% of adults had depression, anxiety, and stress after the onset of COVID-19 in Australia (11).

An overwhelming body of evidence has demonstrated the positive benefits of engaging in adequate physical exercise on improving mental health well-being (12–14). For example, previous review and meta-analysis have shown that appropriate exercising at social isolation may enhance self-efficacy and self-mastery to control and reduce depression (15) and anxiety (16), and may increase self-acceptance to achieving internal goals and satisfactions (17); recreational and home-based group exercise may provide an environment for emotional communication and sharing to relax and relieve mental and emotional stress (18). However, relevant evidence has indicated a high prevalence of physical inactivity among the general population (19), which has worsened due to the outbreak of the pandemic (5). And there is a severe lack of psychological exercise intervention measures (20), and individuals in social isolation have very limited or no access to mental healthcare during the pandemic in most countries (21). To design effective physical exercise promotion programs to help people reap the great benefits of regular physical exercise on mental health well-being during the pandemic, a better understanding of the interrelationship between physical exercise and mental health in the special context is vitally important, especially in a national representative sample. Given that the improvement of mental health may vary as a function of different physical exercise forms, frequencies, intensities, or duration (22, 23), we intended to explore answers for the following questions: what types of physical exercise are beneficial for improving psychological status? Whether all types are equally beneficial for improving mental health, or whether certain forms of physical exercise have advantages over others during the epidemic?

From January 24 to April 22, the Chinese government adopted a series of prevention strategies, such as locking down entire cities and implementing home quarantines and social distancing, and consequently, the epidemic was effectively controlled within 3 months (17, 24). This situation in China may offer a relatively stable research environment and a huge amount of samples to this study due to the essential research question is the relationship between physical exercise and mental health during the epidemic. Therefore, the study aimed to reveal the association between physical exercise and mental health during the outbreak of COVID-19 in China through a large-sample survey. Investigations such as the present study are essential for providing evidence to inform policymakers and guide future policy and program planning to promote physical exercise and improve mental health during periods of public health emergencies.

## Materials and Methods

### Study Population

According to the Chinese Health Commission, which defined the stages of development of the epidemic in China, the peak of the COVID-19 outbreak in China was from January 24 to April 22, 2020. The study recruited Chinese residents living in 31 provinces of China covered by the physical fitness monitoring point system during this period. The study sample included individuals who were not infected with COVID-19 because China has adopted unified quarantine management measures for people infected with COVID-19. The participants were divided into the following 10 groups based on their age: 17 years old and below, 18-24 years old, 25-29 years old, 30-34 years old, 35-39 years old, 40-44 years old, 45-49 years old, 50-54 years old, 55-59 years old, 60 years old and above. The sample size of each province with quotas based on the sampling plan of the sixth national physical fitness monitoring. Furthermore, full ethical approval was obtained from the Wuhan Sports University, and all participants gave informed consent.

### Survey Methodology

This was a nationwide cross-sectional study. The survey content included physical exercise and mental health data, and the participants' gender, age, education level, geographic location (province and city), and social factors, such as occupation and region (towns and villages). The study used a snowball sampling strategy to recruit questionnaire respondents due to the recruitment pool of participants, and a questionnaire was distributed *via* the WeChat and social media platforms with high click-through rate and usage rate of all group. In addition, instructors in social sports in various communities, cities, and provinces participated in questionnaire distribution *via* their working platforms. Through these ways, ensure that residents from different regions of the country participated and to maximize the diversity and representativeness of the population participating in the survey. Within the questionnaire, participants were asked to recall their mental health and physical exercise during the pandemic. The data collection period was from June 20 to July 30, 2020.

Physical exercise data were collected using the Chinese version of the International Physical Activity Questionnaire (IPAQ-C) (21). All data were managed and screened according to standard methods and the guidelines for data processing and analyses of the IPAQ. Individuals were asked to recall the type, intensity, frequency, and duration of various physical exercises they engaged in per day and per week during the COVID-19 outbreak.

Mental health was assessed using the 50-item Self-evaluation Table for Chinese Residents' Mental Health during the Outbreak Peak of COVID-19, which was compiled based on the Symptom Checklist 90 (SCL-90). The Cronbach's alpha coefficient was 0.91, and the test-retest reliability was above 0.7. The internal consistency was 0.87. Individuals were asked to recall the specific influences on their mental health during the pandemic, as well as the specific changes in anxiety, fear, depression, somatization, and stress before and after physical activity. Each item was rated on a five-point Likert scale, ranging from 1 (very slightly or not at all) to 5 (extremely), to assess the extent to which the participants felt that each mental health item pertained to them during the peak of the COVID-19 outbreak. The higher the total score indicated more serious the individual's psychological problems and the lower his or her mental health level.

### Statistical Analysis

All data were analyzed with SPSS software 26.0 (IBM Inst., Chicago, IL, USA). Descriptive statistics such as percentages, 95% CIs, means, and standard deviations were calculated for categorical variables and continuous variables to reflect the demographic characteristics of the survey population. The Kolmogorov-Smirnov test was used to test the normality of continuous variables. Spearman's correlation coefficients were calculated to assess the relationship between the provincial proportions of confirmed COVID-19 cases and the extent of adverse effects on mental health in various provinces and cities. The chi-square test was used to determine the statistical significance of the differences in the proportions of confirmed COVID-19 cases among provinces and the mental health status of participants from different provinces. Regression analysis was conducted to reveal the effect of self-reported physical exercise (i.e., type, duration, and frequency) on reducing mental health burden during the COVID-19 outbreak. Weekly total duration of physical exercise was calculated.

The provincial proportions of confirmed COVID-19 cases were calculated by dividing the total number of confirmed COVID-19 cases (as of 22 April 2020) by the total population (as of the end of 2019) for each of the 31 provinces. The provincial population at the end of 2019 was quoted from the China Statistical Yearbook published by the National Bureau of Statistics of China (2020).

## Results

### Survey Respondents

During the COVID-19 pandemic, a total of 14,715 participants were included in the final analysis (Table 1); these participants were from 31 provincial administrative regions in mainland China (Table 2).

**Table 1:** Basic characteristics of the respondents (*n* = 14,715 participants).

**Category**	**Frequency**	**Percent**	**Mean**	**Standard deviation**
**Gender**				
Male	6,894	47%	1.53	0.50
Female	7,821	53%		
**Age (years)**				
≤ 17	1,258	8.5%	4.93	2.31
18 ~ 24	2,480	16.9%		
25 ~ 29	2,054	14.0%		
30 ~ 34	1,615	11.0%		
35 ~ 39	1,756	11.9%		
40 ~ 44	1,704	11.6%		
45 ~ 49	920	6.3%		
50 ~ 54	1,040	7.1%		
55 ~ 59	828	5.6%		
60 ~ 69	1,060	7.2%		
**Education level**				
Primary school and below (including those with no systematic education)	753	5%		
Junior high school	1,630	11%		
High school (higher vocational school, technical secondary school, and technical school)	1,873	13%	5.25	1.34
Junior college (self-examination, adult education, and promotion)	3,176	22%		
Bachelor's degree	4,909	33%		
Master's degree or above	2,374	16%		
**Urban and rural areas**			1.34	0.48
Cities and towns	8,039	55%		
Rural	6,676	45%		
**Profession**				
Front-line medical staff for epidemic prevention	520	4%		
Front-line epidemic prevention volunteer	599	4%		
Public institution/civil servant/government staff	1,559	11%		
Professional (e.g., teacher/lawyer)	1,538	11%		
Service personnel (e.g., catering waiter/driver/salesperson)	742	5%		
Freelancer (writer/artist/tour guide, etc.)	694	5%	7.94	3.40
Worker (e.g., factory worker/construction worker/city sanitation workers)	674	5%		
Company employee	1,516	11%		
Businessman/employer/salesperson/self-employed	620	4%		
Student	3,005	21%		
Housewife	801	6%		
Farmer/herdsman/fisherman	1,154	8%		
Unemployed/unemployed	997	7%		

**Table 2:** Distribution of respondents across provinces and cities.

**Province**	**Count**	**Province**	**Count**	**Province**	**Count**
Beijing	408	Anhui	518	Guizhou	385
Tianjin	450	Fujian	657	Yunnan	455
Hebei	393	Jiangxi	687	Tibet	301
Shanxi	433	Henan	739	Chongqing	285
Inner Mongolia	336	Hubei	824	Shanxi	359
Liaoning	455	Hunan	780	Gansu	351
Jilin	453	Guangdong	742	Qinghai	389
Heilongjiang	302	Guangxi	483	Ningxia	437
Jiangsu	583	Hainan	489	Xinjiang	240
Zhejiang	406	Sichuan	507		
Shanghai	386	Shandong	482		

### Mental Health Status

During the COVID-19 outbreak, mental health was affected to varying degrees across all groups (Table 3), with somatization (2.416, 95% CI 2.401-2.431), anxiety (2.315, 95% CI 2.300-2.330) and stress (2.218, 95% CI 2.203-2.232) being affected to a greater extent than other aspects of mental health.

**Table 3:** Mental health status during the COVID-19 outbreak.

	**M ± SD**	**Variance**	**95% CI (LL)**	**95% CI (UL)**
Somatization	2.416 ± 0.925	0.855	2.401	2.431
Anxiety	2.315 ± 0.922	0.851	2.300	2.330
Depression	1.879 ± 0.876	0.767	1.865	1.893
Stress	2.218 ± 0.896	0.803	2.203	2.232
Fear	1.818 ± 0.840	0.705	1.805	1.832

*M, mean; SD, standard deviation*.

### Factors Affecting Psychological Status

**Figure 1:**
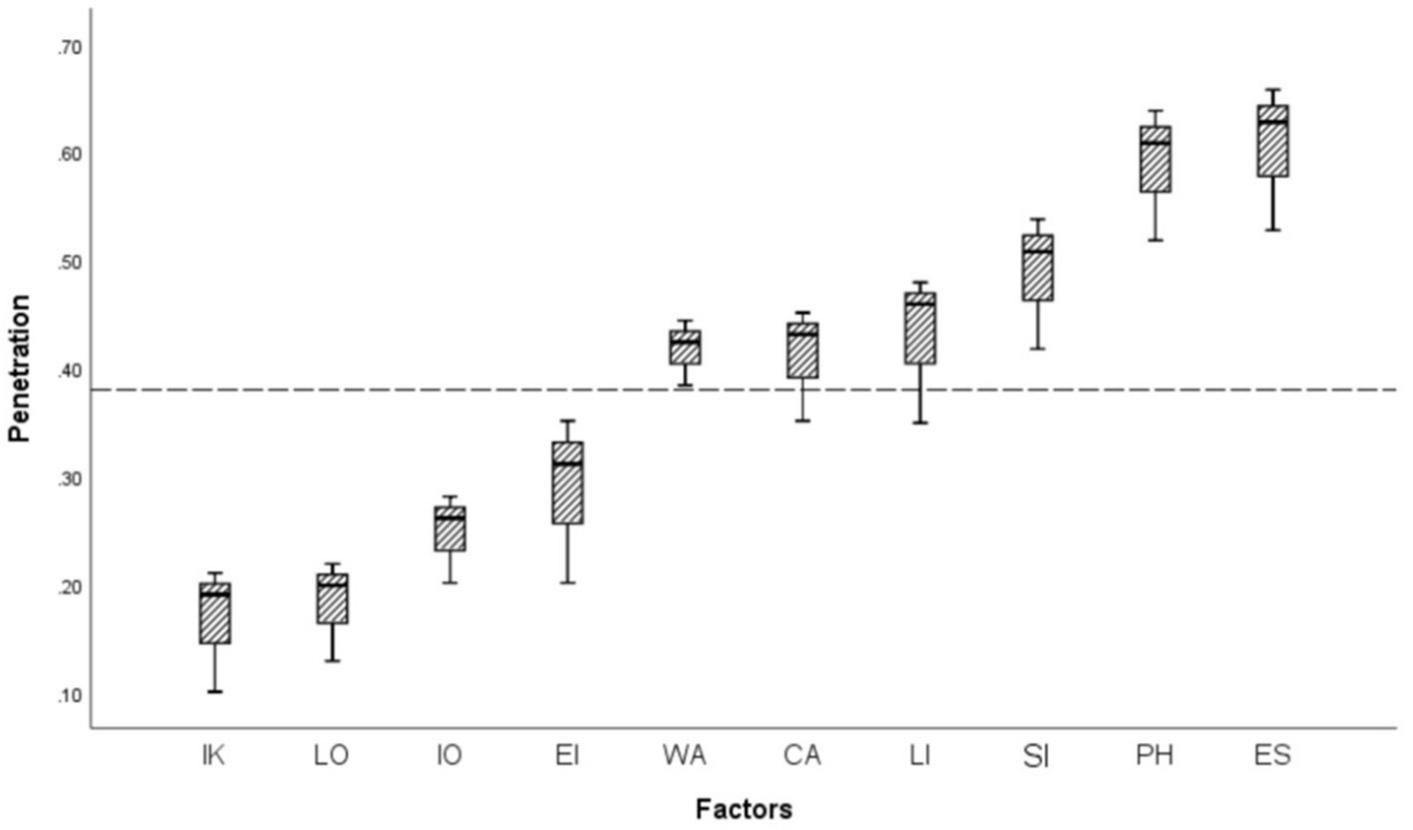
Factors affecting mental health during the COVID-19 outbreak. IK, Insufficient knowledge and understanding of epidemic prevention measures; LO, Lack of effective epidemic prevention equipment; IO, Impact on work processes; EI, Impact on economic income; WA, Worries about being infected; CA, Concerns about students' learning; LI, Limitations in daily life and social interaction; SI, Spread of large amounts of negative information about the epidemic; PH, Prolonged home quarantine; ES, Epidemic severity.

We observed many factors that could cause mental health burden (Figure 1) during the COVID-19 outbreak, with the main factors including epidemic severity (64.77%, 95% CI 58.62-67.64%), prolonged home quarantine (62.84%, 95% CI 57.15-64.25%), spread of large amount of negative information about COVID-19 in the media (54.78%, 95% CI 49.46-58.15%), limitations in daily life and social interaction (46.93%, 95% CI 43.46-48.55%), concerns about students' learning (43.13%, 95% CI 40.26-45.48%), and worries about being infected (42.13%, 95% CI 39.16-45.23%).

### Correlation Analysis of Mental Health Status and Infection Rate in Provinces and Cities

**Figure 2:**
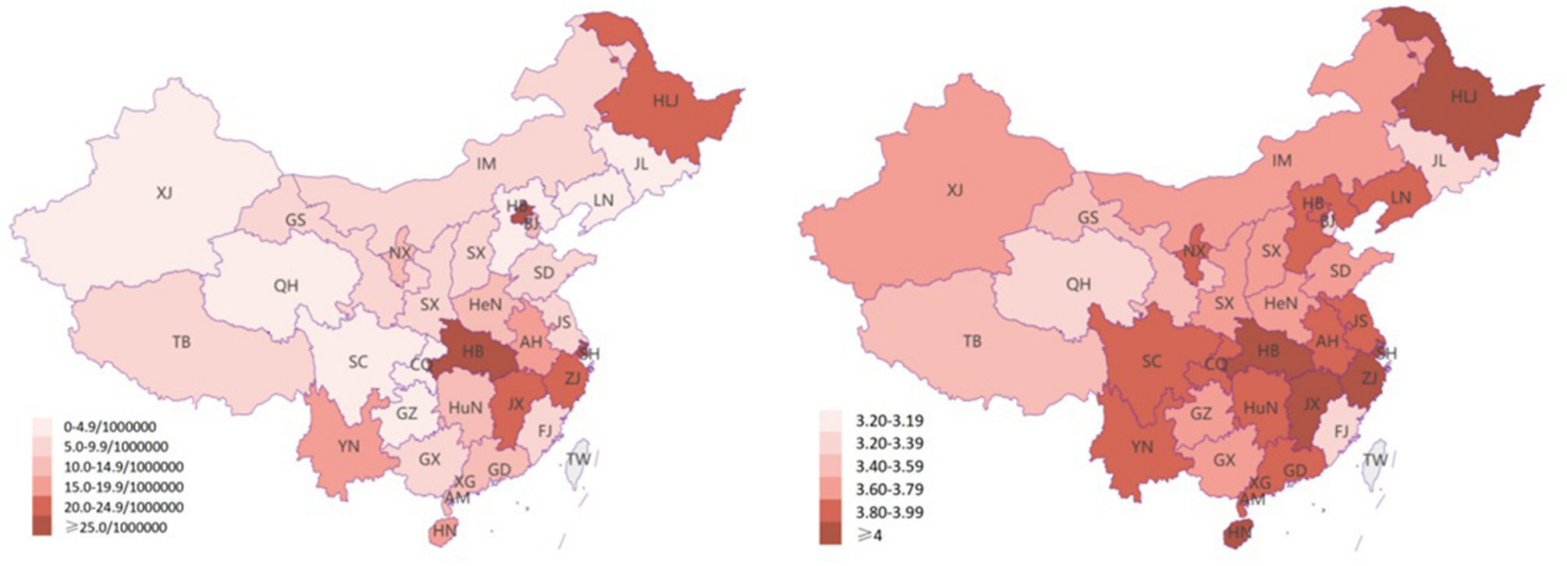
The COVID-19 infection rate and psychological impact in each province and city. The first map shows the provincial proportions of confirmed COVID-19 cases, and the second map shows the psychological impact.

**Figure 3:**
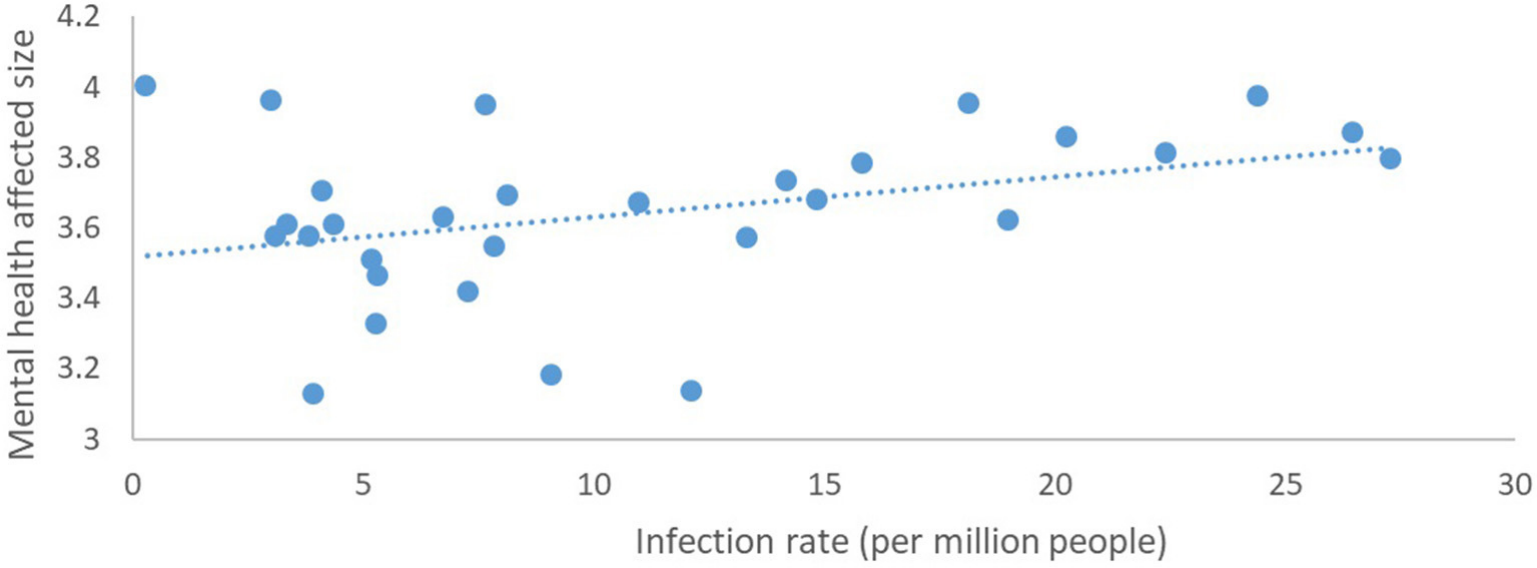
The relationship between the degree of mental health impact and provincial proportions of confirmed COVID-19 cases.

Correlation analysis was carried out on the degree of influence on mental health and the COVID-19 infection rate (per million people), and the Pearson correlation coefficient was used to indicate the degree of the correlation. According to the scatter plot to determine the linear fit of the data, the degree of influence on mental health = 0.0114^*^ infection rate (per million) + 3.5175, and the R-squared value was 0.133. The analysis showed that the correlation value between the degree of the effect on mental health and the provincial proportions of confirmed COVID-19 cases (per million people) (Figure 2) was 0.365 (*p* < 0.05) (Figure 3), indicating a significantly positive correlation between these two variables.

### Analysis of the Influence of Physical Exercise on Mental Health

#### The Positive Influence of Physical Exercise on Mental Health Status

During the outbreak of COVID-19, physical exercise had significant positive effects on reducing mental health burdens, including somatization, anxiety, depression, stress and fear, for all age groups (2.79, 95% CI 3.34-2.13, Table 4); for each type of mental health burden, the correlation with physical exercise was >0.

**Table 4:** The positive influence of physical exercise on mental status.

	**M ± SD**	**Variance**	**95% CI (LL)**	**95% CI (UL)**
Improved somatization	2.09 ± 1.109	1.231	2.54	1.73
Decreased anxiety	3.02 ± 1.112	1.236	3.95	2.06
Decreased depression	2.32 ± 1.117	1.248	2.58	1.69
Decreased stress	2.81 ± 1.113	1.239	3.55	2.6
Decreased fear	3.25 ± 1.103	1.217	3.59	3.03
Improved mental health	2.79 ± 1.049	1.099	3.34	2.13

*M, mean; SD, standard deviation; CV, coefficient of variation*.

#### Influence of Physical Exercise Types on Mental Health

**Figure 4:**
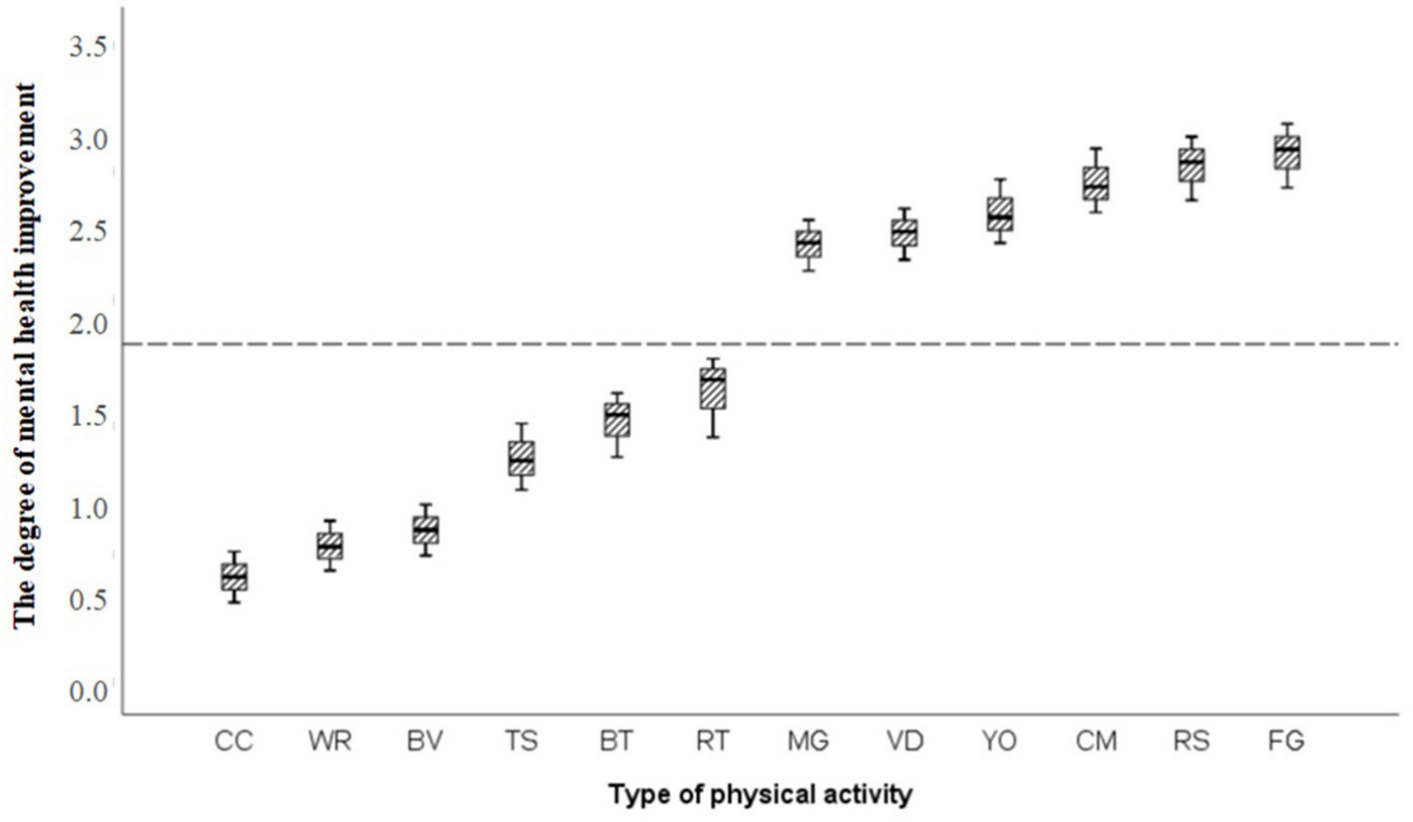
The effect of physical exercise on improving mental health. CC, Chess classes; WR, Walking or running; BV, Basketball, volleyball or football; TS, Taking the stairs; BT, Badminton, tennis or table tennis; RT, Resistance training. MG, Sensory-motor games and whole body vibration; VD, Video dancing; YO, Yoga; CM, Chinese martial arts, Taijiquan and Qigong; RS, Rope skipping or badminton; FG, Family games.

Binary logistic regression was used to analyze the influence of physical exercise types on mental health improvement during the COVID-19 outbreak period. A *P*-value <0.05 indicated that the physical exercise type influenced mental health improvement. The box chart was drawn to show the improvement in mental health with each exercise type and the 95% CI values as the error lines. We observed that specific types of physical exercise were associated with a greater reduction in mental health burden than others during the pandemic (Figure 4), including home-based group entertainment exercise, such as family games (3.151, 95% CI 2.915-3.342), rope skipping and badminton (3.087, 95% CI 2.869-3.192); Chinese traditional sports (2.806, 95% CI 2.694-3.010), such as Chinese martial arts, Taijiquan and Qigong; and popular sports, such as yoga (2.587, 95% CI 2.474-2.742), video dancing (2.431, 95% CI 2.324-2.572), sensory-motor games and whole-body vibration (2.402, 95% CI 2.311-2.502).

#### Influence of Physical Exercise Intensity on Mental Health

**Figure 5:**
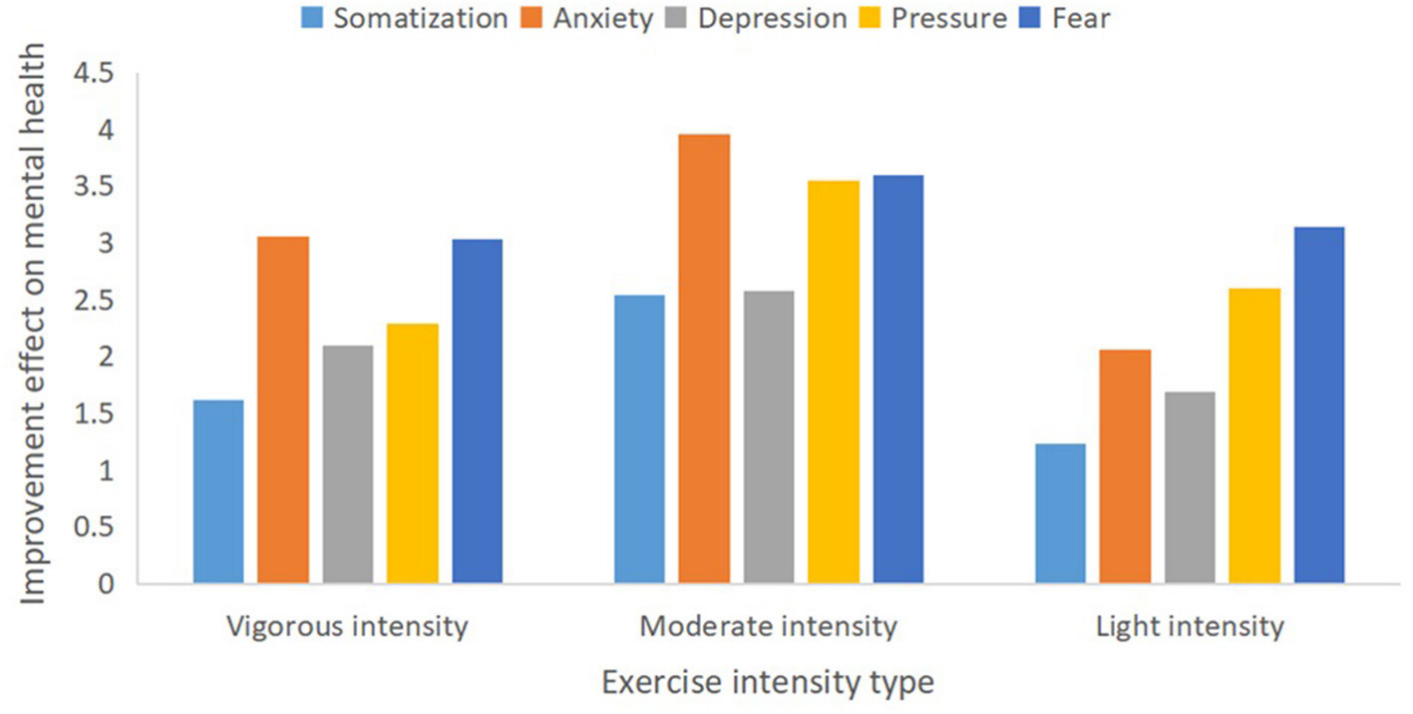
Different physical exercise intensities and mental health improvement.

According to the standards of the International Health Organization (23), physical exercise intensity is divided into vigorous intensity (≥80% of the maximum heart rate, manifested as strenuous activity, shortness of breath, and accelerated heart rate, which can cause significant fatigue), moderate intensity (~60-69% of the maximum heart rate, manifested as slightly increased breathing and heart rate during exercise, no shortness of breath, slight sweating, slight fatigue, and waking up the next day without feeling tired), and light intensity (~35-59% of the maximum heart rate, such as when walking slowly, and feels relaxed). We found that moderate-intensity exercise (3.242, 95% CI 3.01-3.58, *p* < 0.05) was better than both light-intensity (2.56, 95% CI 2.31-2.78, *p* < 0.05)and vigorous-intensity (3.03,95% CI 2.19-3.14, *p* < 0.05)exercise for mental health improvement during the COVID-19 outbreak in China (Figure 5).

**Figure 6:**
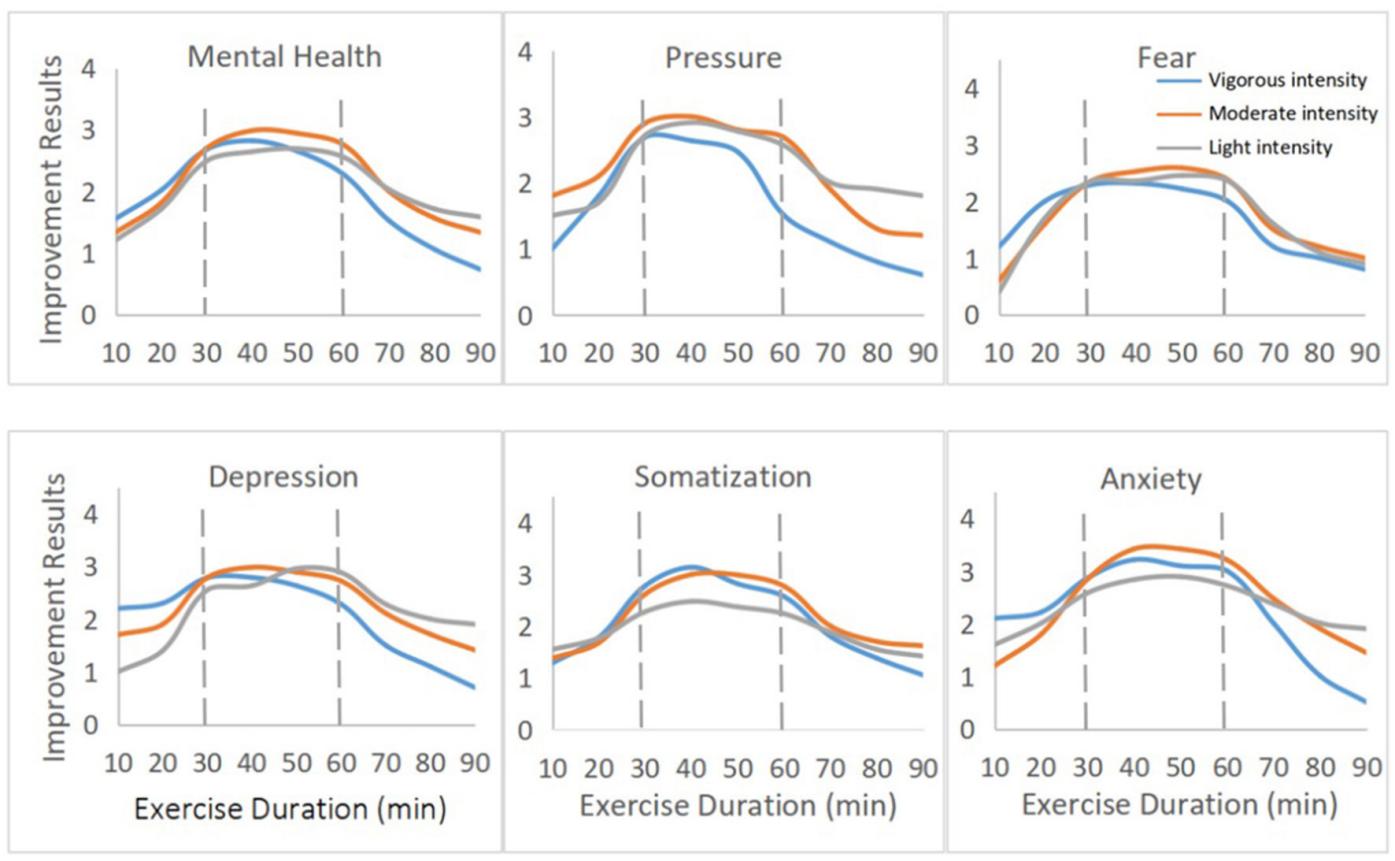
The association between mental health improvement and exercise frequency per week.

**Figure 7:**
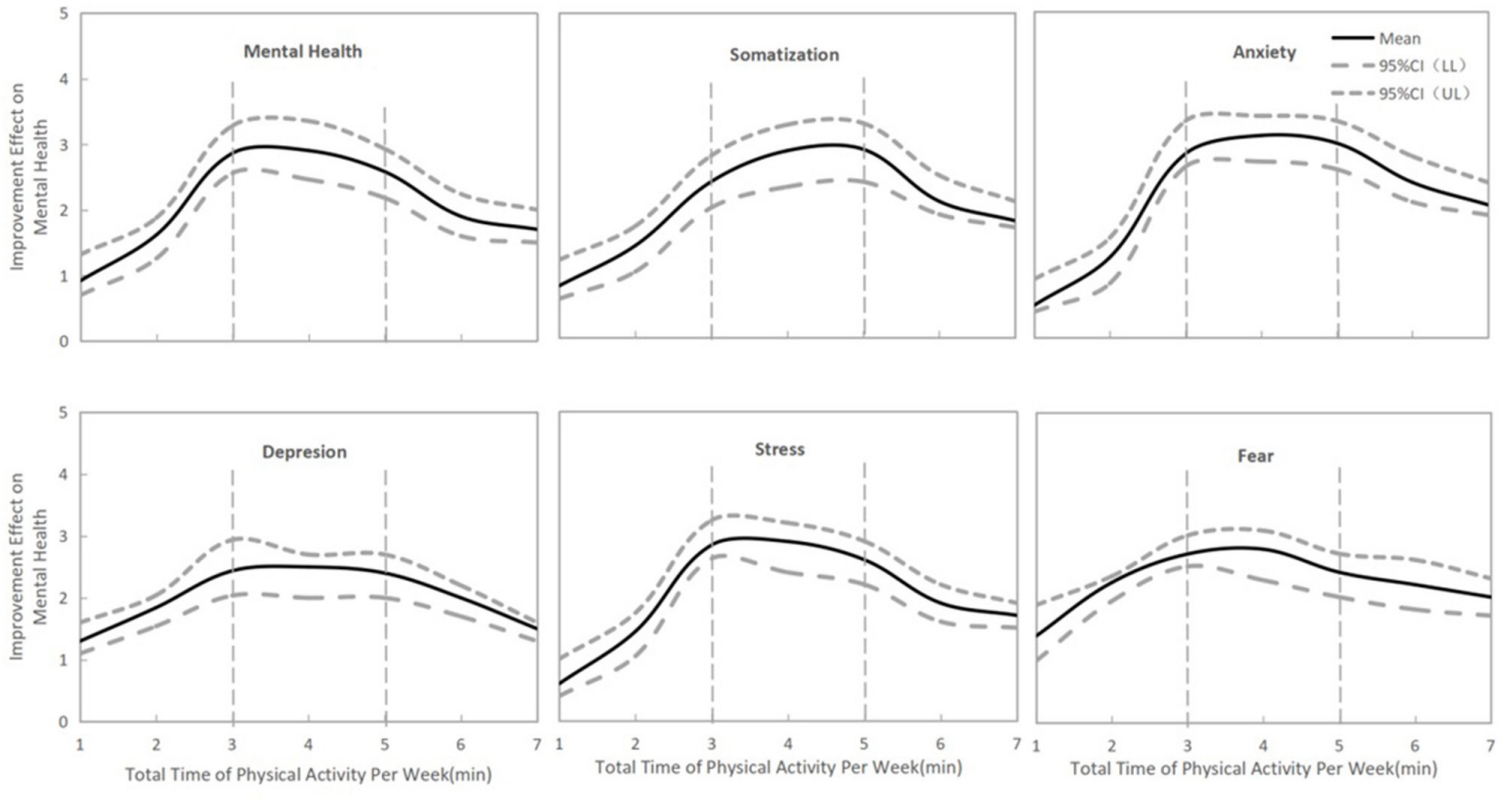
The association between mental health improvement and exercise duration per session.

We observed inverted U-shaped associations between mental health improvement and exercise duration per session (Figure 6) as well as frequency of exercise per week (Figure 7). According to the duration per session of different exercise intensities, the average degree of mental health improvement was fitted, and a trend chart was drawn for all levels of physical exercise intensity. An exercise duration per session between 30 and 60 min (*p* < 0.05) was associated with the best mental health improvement. In general, lower reductions in mental health burden were seen for individuals who engaged in more than 90 min of exercise during the pandemic.

Regarding exercise frequency, three to five times per week was associated with the best mental health improvement (Figure 7, *p* < 0.05). In general, small reductions in mental health burden were seen for individuals who participated in physical exercise fewer than three times per week or more than six times per week.

**Figure 8:**
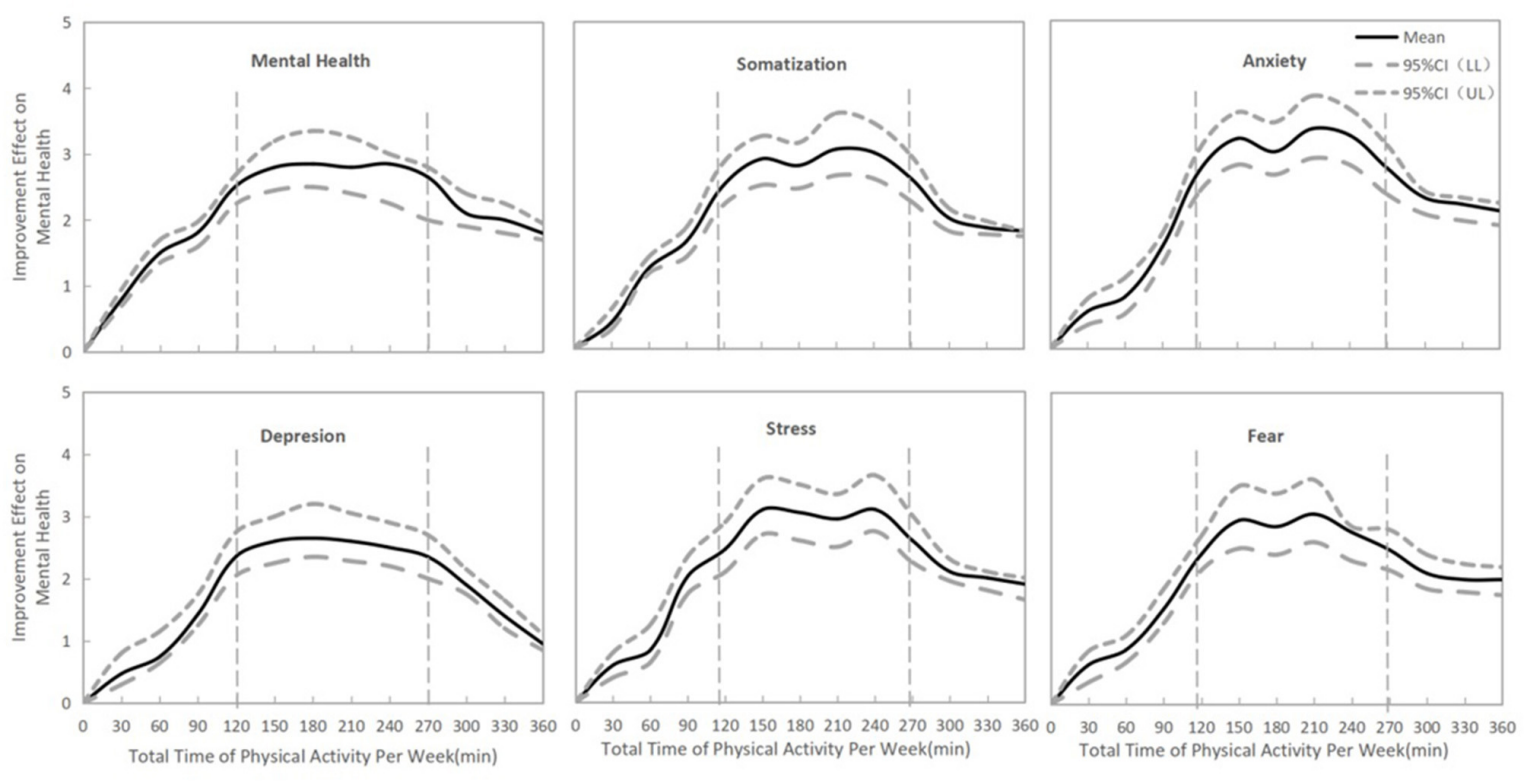
The association between mental health improvement and weekly total physical exercise.

For all types of mental health burden, a total time of physical exercise per week between 120 and 270 min was associated with the best mental health improvement during the COVID-19 outbreak in China (Figure 8, *p* < 0.05).

## Discussion

### Analysis of Mental Health Status

The present study showed that during the COVID-19 outbreak, people in China experienced adverse psychological symptoms, such as anxiety, depression, stress, fear, and somatization. Moreover, we found a positive and significant correlation between provincial proportions of confirmed COVID-19 cases (per million people) and the degree of effects on mental health, and epidemic severity was the most important factor causing mental health burden. Fei Qin et al. (5) also reported a similar correlation (r = 0.501, *p* = 0.004) at the beginning of the outbreak in China.

Overall, the growing numbers of confirmed COVID-19 cases, deaths and the uncertainty of infection could raise stress and anxiety, while depressive and loneliness were likely due to the mandatory social distancing measures during the COVID-19 outbreak (25); all these factors could cause deleterious effects on mental, cardiovascular and immune health (26). Home quarantine also poses considerable financial, psychological and emotional problems for people and might lead to an increase in mood disorders such as panic disorder, anxiety and depression (27). In addition, the COVID-19 outbreak was the first social- media information epidemic (28). With screen time exceeding 4 h per day during the home quarantine of Chinese citizens at the beginning of the COVID-19 outbreak (5), the large amounts of information disseminated by the media was likely to increase public concern about being infected and to induce or exacerbate public anxiety, stress, and other adverse emotions. For example, a survey showed that 54% of respondents rated the mental health impact of the COVID-19 outbreak as moderate or severe, 29% reported moderate to severe anxiety symptoms during the COVID-19 outbreak in China (10). Somatization, anxiety, fear, and stress were higher than normal in front-line medical staff working in epidemic prevention. However, most people did not receive effective physical exercise interventions for mental healthcare during the pandemic (20).

Similar problems occurred worldwide. For example, almost 30% of adults drank more than usual to cope with their psychological depression, anxiety, and stress after the onset of COVID-19 in Australia (11). In Italy, prolonged home quarantine was found to be associated with increased mental health burden, including post-traumatic stress symptoms, avoidance behaviors, and anger (29). In addition, poor mental health states are known to increase the risk of acute respiratory infections (8) and to produce deleterious effects on cardiovascular and immune health (9). Therefore, promoting mental health is important and necessary during the pandemic.

In addition, the United Nations Educational, Scientific and Cultural Organization reported that ~861.7 million students were out of school worldwide due to COVID-19 (13). As the result of the pandemic, the Chinese government implemented a comprehensive school closure strategy. In the first half of 2020, some secondary school students were facing secondary and high school exams and college graduates needed to find jobs in China. The limitations on students' learning increased anxiety and stress among parents and students. However, the timely and comprehensive online learning strategy adopted by the Chinese government somewhat alleviated the stress and anxiety caused by the suspension of offline classes (6). But excessive screen time also generates certain adverse effects. For example, a survey of the National Ministry of Education showed that the myopia rate of primary and secondary students increased by 11.7% during the epidemic prevention period in China (the first half of 2020) (30).

Almost all people are vulnerable to mental health problems in the face of public health emergencies. During the SARS pneumonia (31) and influenza A H1N1 (32) outbreaks, people also experienced a variety of psychological problems in China, such as anxiety, depression, and panic. During the outbreak of COVID-19, the Chinese government established a strong material security system to ensure that people had sufficient epidemic prevention equipment, medical testing and treatment resources, which played an important role in reducing the mental health burden. However, areas that need to be improved to promote psychological health during the outbreak of COVID-19 include determining how to quickly build an authoritative information and media platform to strengthen the effective, accurate and authentic dissemination of various epidemic information and epidemic prevention knowledge, as well as determining how to improve people's home lives during the longer home quarantine period.

### Analysis of the Relationship Between Physical Exercise and Mental Health

The present study showed that all types of physical exercise were associated with significant reductions in different types of mental health issues, such as stress, anxiety, depression, and somatization. Related studies have also reported positive effects of physical exercise in improving negative and unhealthy emotions (5, 33) in different populations during the COVID-19 pandemic. First, self-mastery is a crucial criterion for promoting positive impacts on psychological outcomes (34). Self-efficacy or mastery and psychological control were found to be enhanced when people maintained self-regulation, self-judgment, and self-discipline in engaging in physical exercise during the COVID-19 outbreak period. Exercising at home can increase individuals' sense of control and distract them from negative and worrying thoughts and rumination (9). Second, in the context of social isolation, physical exercise may be one key to increasing self-acceptance and one's sense of competence and to achieving internal goals and satisfaction, contributing to greater positive mental health (35). Third, physical exercise was found to be an effective means of maintaining physical, mental and immune health and reducing socioeconomic burden or medical burden during the epidemic (5). However, few public health guidelines around the world include daily physical exercise routines for people living in varying degrees of quarantine during the pandemic (17). Individuals who were socially isolated during the epidemic were found to have no access to mental healthcare in the vast majority of cases (9). Overall, on the one hand, the government's efforts to improve physical exercise intervention for mental health during the epidemic should be strengthened. On the other hand, individuals' motivation to exercise should be stimulated to increase their desire to exercise and the awareness of the benefits of exercise for physical and mental health improvement among the general public. Finally, potential mechanisms for exercise intervention, monitoring and prevention mechanisms for improving metal health should be systematically developed during public health emergencies. Such measures are of great importance for strengthening the effect of physical exercise on mental health improvement.

Most people experienced restrictions on physical exercise and were forced to change their exercise methods during theCOVID-19 outbreak (16). The study also observed that specific types, durations, and frequencies of physical exercise might be more effective than others for mental health improvement during the pandemic period.

Firstly, recreational and home-based group exercise can provide individuals with an environment for emotional communication, sharing and support from multiple individuals, which is of great benefit to mental and emotional relaxation and stress relief (18). Therefore, group-exercise formats that involve activities with family members or the use of various forms of network visualization with friends or organizations may be more effective than individual exercise in reducing mental health burden.

Secondly, traditional Chinese sports and trendy forms of exercise may be associated with a better reduction in mental health burden. Related studies have reported that traditional Chinese sports, such as Chinese martial arts, Taijiquan and Qigong, which involve a sequence of movements and postures with the regulation of the breath rhythm and pattern, musculoskeletal stretching and relaxation, may be potentially useful for the prevention, treatment and rehabilitation of COVID-19, as well as for emotion control, stress reduction, mental improvement, and enhanced immune function (36–38). Related studies have also shown that popular sports have the potential to play a role in metal health improvement. For example, ~20 min of the moderate-intensity exergame Zumba Fitness significantly reduced anxiety in healthy young women (16). An 8-week video game intervention (i.e., dance, postural control, coordination, and walking training) was found to have a significant improvement in anxiety (39). In addition, sensory-motor activities were noted as safe, fun and valuable means of increasing girls' motivation to participate in physical exercise while staying at home (9). In general, the effectiveness of physical exercise in improving mental health depends on the degree of internalization of the behavior. Exercising at home may be accompanied by higher self-esteem and lower psychological ill-being when people are free to choose the exercise type, schedule, frequency and intensity that are consistent and assimilated with the individual's goals and interests, personal characteristics, abilities and identity (13). Therefore, according to public needs, the establishment of a library of traditional Chinese sports and trendy home-based exercises would be an effective measure to improve mental health during home quarantine and social distancing due to COVID-19.

Thirdly, related studies have reported that both the intensity of exercise (relative load lifted) and physiological adaptation (muscle strength gained) were significantly related to the magnitude of the depression response (40), and moderate-intensity exercising may be accompanied by health benefits including effective disease prevention and the maintenance of psychological, muscular, metabolic, and cardiovascular health during home isolation (41). On the one hand, based mainly on the J–shaped relationship between physical exercise intensity and muscle immunity (42), medium-intensity exercise may generate more health benefits for the immune system and the body's antiviral defenses (2). However, prolonged, acute and vigorous intensity may suppress immune system function, leading to upper respiratory tract infections and the appearance of latent viral reactivation (43, 44). On the other hand, based mainly on the U-shaped relationships between physical exercise intensity and mental health burden, moderate-intensity exercising may be associated with better mental health improvement than strenuous exercise (25).

Finally, we observed that moderate-intensity exercise lasting 30-60 min per session, a total of 120-270 min of exercise per week and exercise three to five times per week may have better effects on mental health during the COVID-19 outbreak period in China. The International Health Organization recommended that healthy members of the population engage in a cumulative total of 150 min of moderate-intensity aerobic exercise (no <30 min in a single session) or 75 min of high-intensity exercise per week (22). A study also showed that moderate-intensity exercise 30-60 min duration (peaking at ~45 min) per session performed 3-5 times per week was associated with better psychological improvement than other forms of exercise. A worse mental health burden was seen for individuals who exercised more than 23 times per month, more than 6 h per week or more than 2 h per session (22). During the COVID-19 outbreak, home quarantine and social distancing led to severe restrictions on exercise types and facilities and a general lack of comprehensive exercise monitoring, guidance and atmosphere (6). Our investigation showed that more than 90 min of exercise per session and more than 300 min of exercise per week were associated with a significantly lower effect on psychological improvement. Therefore, significantly controlling the duration and frequency of physical exercise may be meaningful and useful for reducing the mental health burden during home quarantine.

### Implications and Limitations of the Study

Our study has several limitations. Firstly, due to the large population of China, the population of different provinces and cities also varies. The questionnaires were distributed through community social platforms, WeChat and other Internet platforms in each province and city to ensure that residents from different regions of the country participated and to maximize the diversity and representativeness of the population participating in the survey. However, because it was an online survey, there was certain unevenness in the participants' age and regional distribution, with relatively more student participants (18-25 years old) and fewer older adults over 60 years old as well as more participants from Hubei and Hunan and fewer participants from Xinjiang and Heilongjiang. Secondly, although we chose the WHO-approved IPAQ-S and the SCL-90, which have high reliability and validity, the use of the participants' self-reported recall of physical exercise and mental health status during the pandemic might not have been as accurate as an intensity detection instrument test. Thirdly, it is necessary to conduct a comparative analysis of mental health status between those who participated in physical exercise and those who did not participate during the pandemic and to further reveal the in-depth effects of physical exercise on mental health, as well as the changing trends in psychological conditions with the continuous development of the epidemic and the extension of home quarantine. Finally, an important next step in our research is to further identify the comprehensive effect of exercise and the exercise intervention mechanism to promote psychological health and prevent COVID-19 infection. Despite these limitations, this study investigated the association between physical exercise and mental health in 31 provinces of China during the COVID-19 pandemic phase, and the results could guide future policies and planning to enhance physical exercise and promote mental health during public health emergencies.

## Conclusion

During the COVID-19 outbreak, Chinese residents showed severe psychological burden, including anxiety, depression, stress, fear, somatization and other mental health burdens. The degree of mental health burden was significantly and positively correlated with the provincial proportions of confirmed COVID-19 cases. The identified factors which severely affected mental health are (1) the severity of epidemic at the national and local levels, (2) the long-term home quarantine, (4) the spread of a large amount of negative information about COVID-19 in the media, (4) limitations in daily life and social interaction, (5) concerns about students' learning, and (6) worries about being infected. All types of physical exercise were significantly associated with improvement in self-reported mental health during the COVID-19 outbreak. Specific types, intensities, durations, and frequencies of physical exercise might be more effective than others for reducing the mental health burden during home quarantine and social distancing. The largest associations were seen for home-based group entertainment exercise, Chinese traditional sports, and popular sports, as well as exercise with a duration of 30-60 min, exercise at frequencies of three to five times per week and a total of 120-270 min of moderate-intensity exercise per week during the COVID-19 outbreak.

## Data Availability Statement

The original contributions presented in the study are included in the article/supplementary material, further inquiries can be directed to the corresponding author/s.

## Author Contributions

YM and YN: conceptualization. YW and YN: methodology and writing—original draft preparation. YW, YM, and JZ: formal analysis. JL, TL, CZ, and CL: investigation. YN, YM, and JZ: writing—review and editing. YW: project administration. All authors made relevant contributions to and approved the final manuscript.

## Conflict of Interest

The authors declare that the research was conducted in the absence of any commercial or financial relationships that could be construed as a potential conflict of interest.

## Publisher's Note

All claims expressed in this article are solely those of the authors and do not necessarily represent those of their affiliated organizations, or those of the publisher, the editors and the reviewers. Any product that may be evaluated in this article, or claim that may be made by its manufacturer, is not guaranteed or endorsed by the publisher.
